# Improved estimation of the cardiac global function using combined long and short axis MRI images of the heart

**DOI:** 10.1186/s12938-016-0156-3

**Published:** 2016-04-27

**Authors:** Hossam El-Rewaidy, Ahmed S. Fahmy

**Affiliations:** Systems and Biomedical Engineering Department, Cairo University, Cairo, 12613 Egypt; Center for Informatics Science, Nile University, Cairo, 12588 Egypt

**Keywords:** Cardiac function, Volume estimation, Geometric models, MRI

## Abstract

**Background:**

Estimating the left ventricular (LV) volumes at the different cardiac phases is necessary for evaluating the cardiac global function. In cardiac magnetic resonance imaging, accurate estimation of the LV volumes requires the processing a relatively large number of parallel short-axis cross-sectional images of the LV (typically from 9 to 12). Nevertheless, it is inevitable sometimes to estimate the volume from a small number of cross-sectional images, which can lead to a significant reduction of the volume estimation accuracy. This usually encountered when a number of cross-sectional images are excluded from analysis due to patient motion artifacts. In some other cases, the number of image acquisitions is reduced to accommodate patients who cannot withstand long scan times or multiple breath-holds. Therefore, it is required to improve the accuracy of estimating the LV volume from a reduced number of acquisitions.

**Methods:**

In this work, we propose a method for accurately estimating the LV volume from a small number of images. The method combines short-axis (SAX) and long axis (LAX) cross sectional views of the heart to accurately estimate the LV volumes. In this method, the LV is divided into a set of consecutive chunks and a simple geometric model is then used to calculate the volume of each chunk. Validation and performance evaluation of the proposed method is achieved using real MRI datasets (25 patients) in addition to CT-based phantoms of human hearts.

**Results:**

The results show a better performance of the proposed method relative to the other available techniques. It is shown that, at the same number of cross-sectional images, the volume calculation error is significantly lower than that of current methods. In addition, the experiments show that the results of the proposed model are reproducible despite variable orientations of the imaged cross-sections.

**Conclusion:**

A new method for calculating the LV volume from a set of SAX and LAX MR images has been developed. The proposed method is based on fusing the SAX and LAX segmented contours to accurately estimate the LV volume from a small number of images. The method was tested using simulated and real MRI datasets and the results showed improved accuracy of estimating the LV volume from small number of images.

## Background

Accurate calculation of the volumes enclosed by the left ventricular (LV) surfaces is required to assess the global functional parameters of the heart [1–4]. Cine Magnetic Resonance Imaging (MRI) has become the reference standard for the assessment of the LV volume and global function [5, 6]. Current clinical protocols include the acquisition of a stack of parallel 2D short-axis (SAX) views, or slices, of the heart from base to apex using standard MRI pulse sequences. Nine to twelve consecutive SAX slices are usually acquired and used to calculate the LV volume. The process begins with delineating the LV endocardium and epicardium contours in all slices [7]. Then, a geometric model that uses these contours to approximate the shape of the heart is used to calculate the LV volumes. This process is repeated for the end-diastole and end-systole phases of the cardiac cycle to calculate differential parameters such as the ejection fraction. It is worth noting that the acquisition of each slice requires the patient not to move and hold his/her breath for few seconds until a cross-section is imaged. Patient motion during the scan and/or failure to properly perform the breath-hold, can lead to severe distortion of the acquired images. This means that, in some cases, it is inevitable to estimate the volume from small number of slices. As will be shown below, this leads to reducing the accuracy of estimating the LV volume. The most widely used-method for calculating the myocardium volume from number of parallel SAX contours is the modified Simpson’s (mSimp) method [8–11]. In mSimp method, the LV volume is approximated by a number of parallel discs. The number of discs is equal to the number of the acquired SAX slices, *N*. The volume, $v_{i}$, of the *i*th disc in the stack is estimated as follows,

1 $$ v_{i} = A_{i} \cdot \left( {t + l} \right), $$

where, $i = 1, 2, \ldots , N; A_{i}$ is the area enclosed by the myocardium contour in the *i*th slice; t is the slice thickness; and l is the inter-slice gap. The total volume is then calculated by taking the summation over all discs. When the number of slices, *N*, is sufficiently large, the mSimp method provides accurate and reliable results even at LV shape anomalies [11]. Nevertheless, the performance of the mSimp method is significantly impacted when the number of the SAX slices decreases due to the inaccurate approximation of large LV segments using simple discs. To avoid these inaccuracies, several models have been proposed to calculate the LV volume from a few planar views of the heart [12–14]. The models assume simplified geometric LV shapes such as ellipsoids and concatenated cylinders and hemispheres. While these models were originally proposed for analyzing echocardiography images, attempts of applying these models to MRI data have been reported by Thiele et al. [14]. However, the accuracy of these models is very limited due to the over-simplification of the cardiac shape that is not valid especially in patients with cardiac anomalies [14, 15].

In this work, we propose a simple geometric model that can be used to estimate the LV volume from a few number of slices; i.e. image acquisitions. The model incorporates information from SAX and long axial (LAX) views to better estimate the shape of the LV at the inter-slice gaps. In the next section, a derivation of the model equations is presented and it will be shown that the volume can be calculated from a simple equation that includes calculating simple geometric parameters such as the areas enclosed by the SAX and LAX contours and the angle between the LAX and SAX planes. The proposed model is validated using 3D cardiac surface generated from Computed Tomography (CT) acquisitions from five human subjects. In addition, real cardiac MRI datasets from twenty five patients have been used to evaluate the accuracy of the proposed method relative to other existing methods.

## Methods

In the methods described below, it is assumed that the volume of the heart is to be estimated from *N* SAX slices and one LAX slice. Our default LAX orientation is the four-chamber view of the heart; i.e. horizontal LAX. Nevertheless, the effect of changing this orientation will be studied as discussed in “Results and discussion” section. The proposed methodology is identical for calculating the volume enclosed by the epicardium and the volume enclosed by the endocardium at any timeframe. Therefore, for simplicity, we will use the general terms of myocardium contours and cardiac volume when discussing calculating the volume enclosed by a set of contours (epicardium or endocardium) at a specific timeframe.

### Problem formulation

**Fig. 1:**
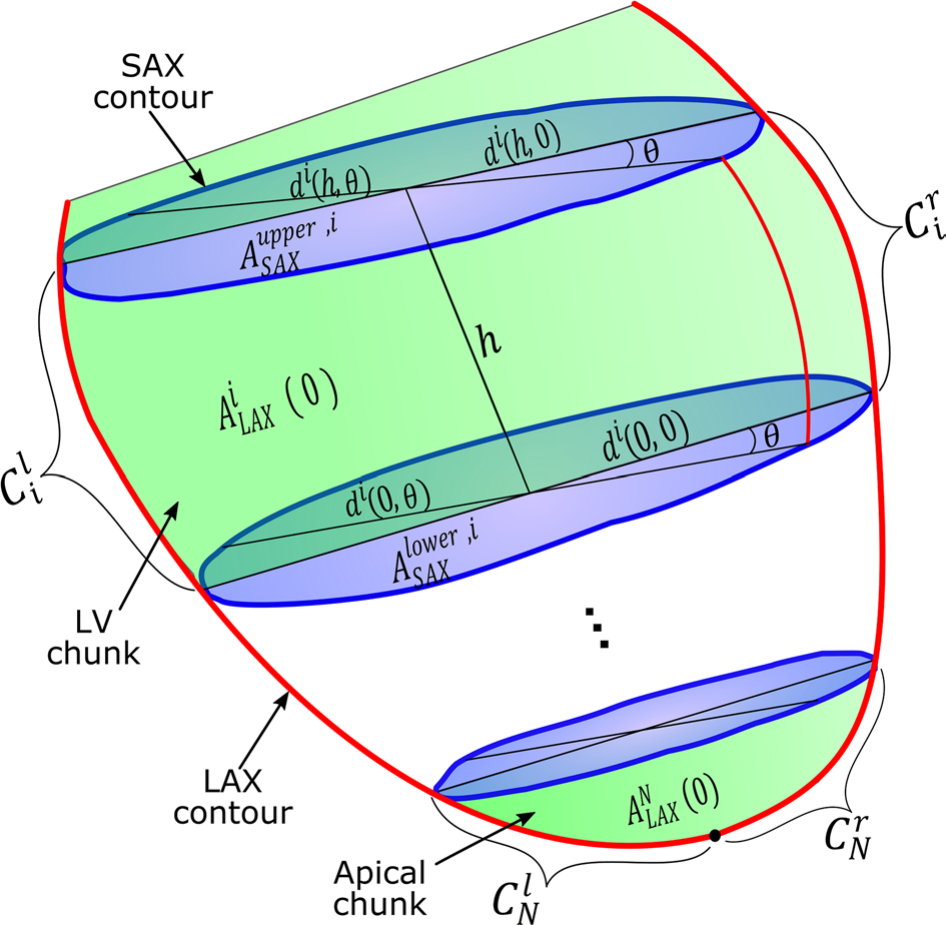
3D schematic plot for the LV showing the SAX contours (*blue*) and the LAX contour (*red*). A number of *N* SAX image planes (*purple areas*) can divide the LV into *N* chunks. *Green* areas annotate the LAX cross-sectional area of the different chunks

Given a number, $N$, of SAX slices and one LAX slice, the myocardium boundaries are delineated to obtain a set of *N* SAX and one LAX contour respectively. Ignoring delineation errors and misregistration due to different levels of breath-holds, these contours can be thought of as a coarse grid representing the intersection between the different image planes and the myocardium surface. It is therefore required to calculate the cardiac volume enclosed by the myocardium surface represented by these contours. As can be seen in Fig. 1, a number of *N* parallel SAX planes can virtually divide the heart into *N* chunks (ignoring the part above the most-basal plane). The plane of the LAX contour intersects with the contour of the upper and lower surfaces of the *i*th chunk and results in a line segments of length $d^{i} \left( {h,0} \right)$ and *d*^*i*^(0, 0), respectively, where *h* is the height of the chunk from the lower surface to the upper.

In general, within the *i*th chunk, the diameter of the upper and lower surfaces at any given angle, $\theta$, are denoted by *d*^*i*^(*h*, *θ*) and *d*^*i*^(0, *θ*), respectively, where *θ* is measured from the plane containing the LAX contour. To account for the unsymmetrical shape of the LAX contour, the right and left parts of the LAX contour within the *i*th chunk are denoted by, *C*_*i*_^*r*^ and $C_{i}^{l}$, respectively. We further define $A_{LAX}^{i}$ (0) as the area enclosed by the curves *d*^*i*^(0, 0), *C*_*i*_^*r*^, *d*^*i*^(*h*, 0), and *C*_*i*_^*l*^. As can be shown in Fig. 1, the area below the most apical slice, $A_{LAX}^{N}$ (0), is enclosed by two curves only: $d^{N} \left( {0, 0} \right),C_{N}^{r}$, and $C_{N}^{l}$. For all the myocardium chunks, $A_{LAX}^{i}$ (0) is numerically calculated by computing the area of a polygon formed by the points on the surrounding curves.

Having defined the basic quantities that are used in the proposed method, the following section describes a simple geometric model that can be used to estimate the cardiac volume of the *i*th chunk from the contour areas, $A_{LAX}^{i}$ (0), and diameters, *d*^*i*^(*h*, 0) and *d*^*i*^(0, 0). Adding the volumes of all chunks yields the required total cardiac volume.

### Cross-sectional modeling using equivalent trapezoids

To simplify the volume calculations, a simple trapezoid is used to approximate the shape of any given long-axial cross-section of an LV chunk. For a given chunk, *i*, all modeling trapezoids are assumed to have the same height, *h*_*i*_, but different lengths of the upper and lower sides depending on the orientation of the LAX plane. For a LAX plane making angle *θ,* with the acquired LAX image plane, upper, *d*^*i*^(*h*, *θ*) and lower, $d^{i} \left( {0,\theta } \right)$, sides of its modeling trapezoid is calculated from the line segments representing the intersection between this LAX plane and the upper and lower SAX contours. The trapezoid height, *h*_*i*_, can be calculated by setting the trapezoid area equal to the cross-sectional area $A_{LAX}^{i}$ (0) described above. That is,

2 $$ h_{i} = \frac{{2 A_{LAX}^{i} \left( 0 \right)}}{{d^{i} \left( {h,0} \right) + d^{i} \left( {0,0} \right)}} $$

For any virtual LAX plane intersecting the *i*th chunk and making an angle, *θ*, with the acquired LAX plane, the area of intersection, $A_{LAX}^{i}$ (*θ*), may also be represented by a trapezoid of height, *h*_*i*_, and thus can be estimated by,

3 $$ A_{LAX}^{i} \left( \theta \right) = \frac{{d^{i} \left( {h,\theta } \right) + d^{i} \left( {0,\theta } \right)}}{2} h_{i} $$

Substituting from Eqs. (2) and (3), the area of the equivalent trapezoid at any angle *θ* can be written in terms of *A*_*LAX*_(0, *i*) as follows,

4 $$ A_{LAX}^{i} \left( \theta \right) = \frac{{d^{i} \left( {h,\theta } \right) + d^{i} \left( {0,\theta } \right)}}{{d^{i} \left( {h,0} \right) + d^{i} \left( {0,0} \right)}} A_{LAX}^{i} \left( 0 \right) $$

**Fig. 2:**
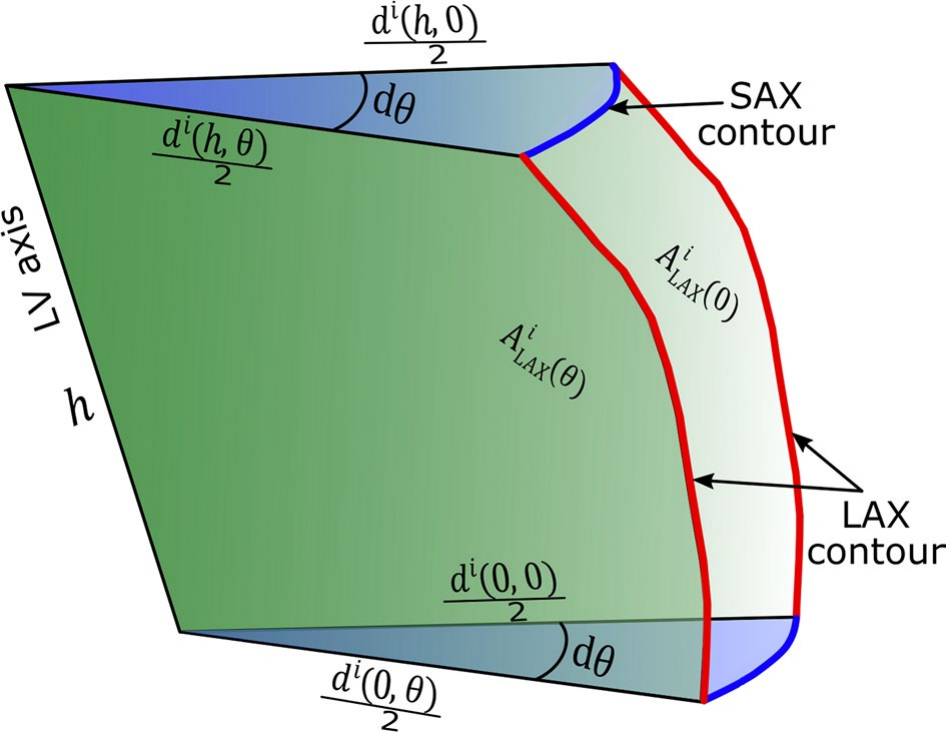
Rotation of a half LAX slice area around the axis of the LV chunk, *h*, with infinitesimal angle, *dθ*, results into a wedge-like shape. Its volume can be determined knowing the rotated area, the distance from the axis to the LAX contour segment, and the rotation angle

If the equivalent trapezoid is rotated with infinitesimal angle, *dθ*, a wedge-like structure is obtained (as shown in Fig. 2) with volume given by,

5 $$ V_{wedge} \left( {\theta ,i} \right) = \frac{{A_{LAX}^{i} \left( \theta \right)}}{2} \times \frac{{\left( {d^{i} \left( {h,\theta } \right) + d^{i} \left( {0,\theta } \right)} \right)/2}}{2} d\theta $$

That is, the volume of the *i*th chunk, $V_{i}$, can be obtained by integrating Eq. (5) from *θ* equal zero to 2π. Substituting from Eq. (4) into (5), it can be shown that,

6 $$ V_{i} = \frac{{0.5 A_{LAX}^{i} \left( 0 \right)}}{{d^{i} \left( {h,0} \right) + d^{i} \left( {0,0} \right)}}\mathop \smallint \limits_{0}^{\pi } \left( {\frac{{d^{i} \left( {h,\theta } \right) + d^{i} \left( {0,\theta } \right)}}{2}} \right)^{2} d\theta $$

Since the SAX contours are available, the diameters *d*^*i*^(*h*, *θ*) and $d^{i} \left( {0,\theta } \right)$ can be readily calculated and the integration in Eq. (6) can be numerically solved. Observing that the integration in Eq. (6) is done over the square of the mean diameter at angle, *θ*, i.e., $d_{mean}^{i} \left( \theta \right) \equiv \frac{{d^{i} \left( {h,\theta } \right) + d^{i} \left( {0,\theta } \right)}}{2}$, then it can be approximated by double the area of a virtual SAX contour with diameter $d_{mean}^{i}$ (*θ*). The area of this virtual contour can be further approximated by the average area of the upper and lower SAX contours; that is,

7 $$ V_{i} \cong \frac{{A_{LAX}^{i} \left( 0 \right) }}{{d^{i} \left( {h,0} \right) + d^{i} \left( {0,0} \right)}}\left( {A_{SAX}^{upper,i} + A_{SAX}^{lower,i} } \right) $$

It is worth noting that, in the most apical chunk (at *i* = *N*), the lower base of the chunk is a single point representing the cardiac apex. That is, the LAX cross-section is approximated by a triangle where the values of *d*^*N*^(0, 0) and $A_{SAX}^{lower,N}$ are set to zero. That is, the volume of the most-apical chunk is calculated using the following equation,

8 $$ V_{N} = \frac{{A_{LAX}^{N} \left( 0 \right) \cdot A_{SAX}^{upper,N} }}{{2 d^{N} \left( {h,0} \right)}} $$

Equation (7) can also be used to calculate the LV volume represented by the LAX contour segments that extend above the most-basal SAX slice (as shown in Fig. 1). First, these free LAX contour segments are used to define a virtual chunk above the most basal SAX plane with volume, *V*_0_. Then, the volume of this virtual chunk is calculated by respectively setting the area *A*_*SAX*_^*upper*,0^ and the diameter *d*^0^(*h*, 0) equal to $A_{SAX}^{lower,0}$ and *d*^0^(0, 0). It can be shown that this approximation results in a volume of a virtual chunk with identical upper and lower surfaces and height equal to the average heights of the two LAX segments extending above the most basal plane. It is worth noting that this volume is excluded from the calculations because there is no reported standard method, and thus a ground truth, for calculating it. It is worth noting that the misregistration between SAX and LAX slices can be corrected by various intensity and contour based methods (as proposed by [16, 17]). Nevertheless, due to imperfect segmentation of the myocardium boundaries in both LAX and SAX images, slight misalignment of the contours causes the LAX contour not to be intersecting with each SAX contour in exactly two points. This gives two possible values for the LV diameter, *d*^*i*^(*h*, 0) and $d^{i} \left( {0,0} \right)$. In this work, the diameters *d*^*i*^(*h*, 0) and *d*^*i*^(0, 0) are calculated from the LAX contours. This is because the LAX slices are less prone to the boundary blurring caused by the partial volume effects and thus the LAX contours are usually more accurate in delineating the LV especially at the apex. Having calculated the cardiac volume for every chunk, the total volume can be then calculated as,

9 $$ Vol = \mathop \sum \limits_{i = 1}^{N} V_{i} $$

### Oblique LAX

**Fig. 3:**
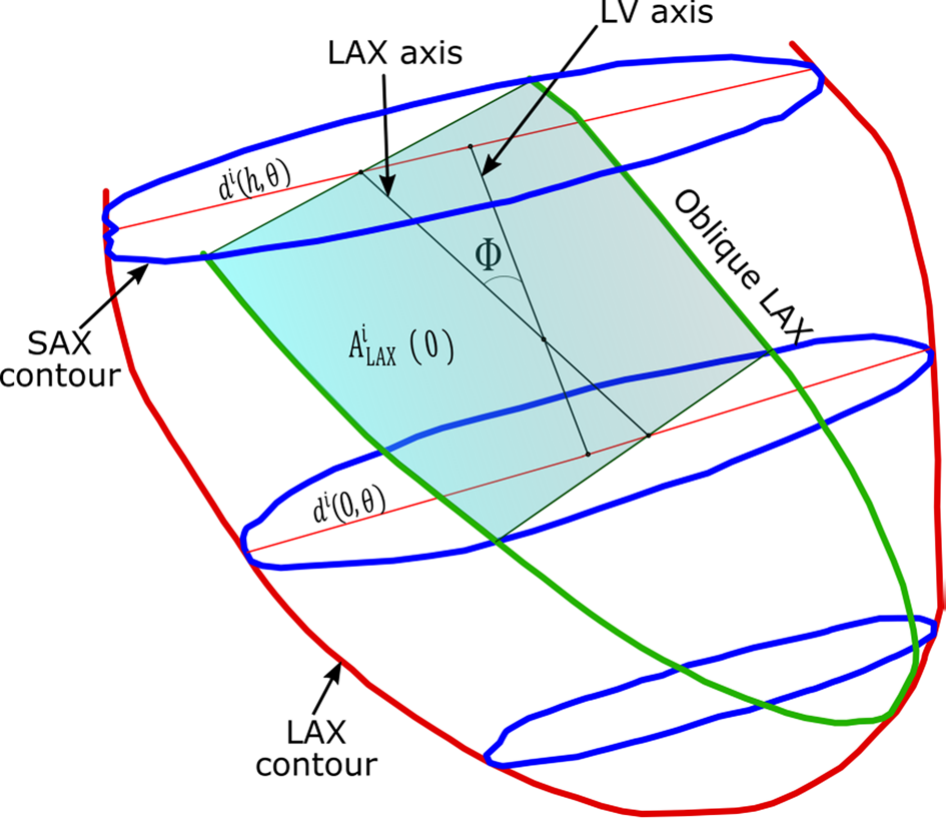
Oblique LAX contour (in *green*) generates a larger intersection area with the cardiac chunk. The correction factor of such area depends on the inclination angle (*Φ*) between the axis of LV and the oblique LAX plane

In practice, the plane of the LAX slice is not perfectly selected perpendicular to the acquired stack of the SAX slices (as shown in Fig. 3). This oblique orientation results in a larger apparent area of the LAX slice and thus the calculated area of the LAX contour, $A_{LAX}^{i}$ (0), should be compensated to account for this factor. One simple solution is to replace $A_{LAX}^{i}$ (0) with a corrected area, $A_{LAX}^{\prime i}$ (0) given by,

10 $$A_{LAX}^{\prime i} (0) = A_{LAX}^{i} \left( 0 \right) \cos \left( {\varPhi_{i} } \right) $$

where *Φ*_*i*_ is the angle between the line connecting the center-of-mass points of the SAX contours forming the chunk and the LAX image plane.

### Model validation using CT-based phantoms

**Fig. 4:**
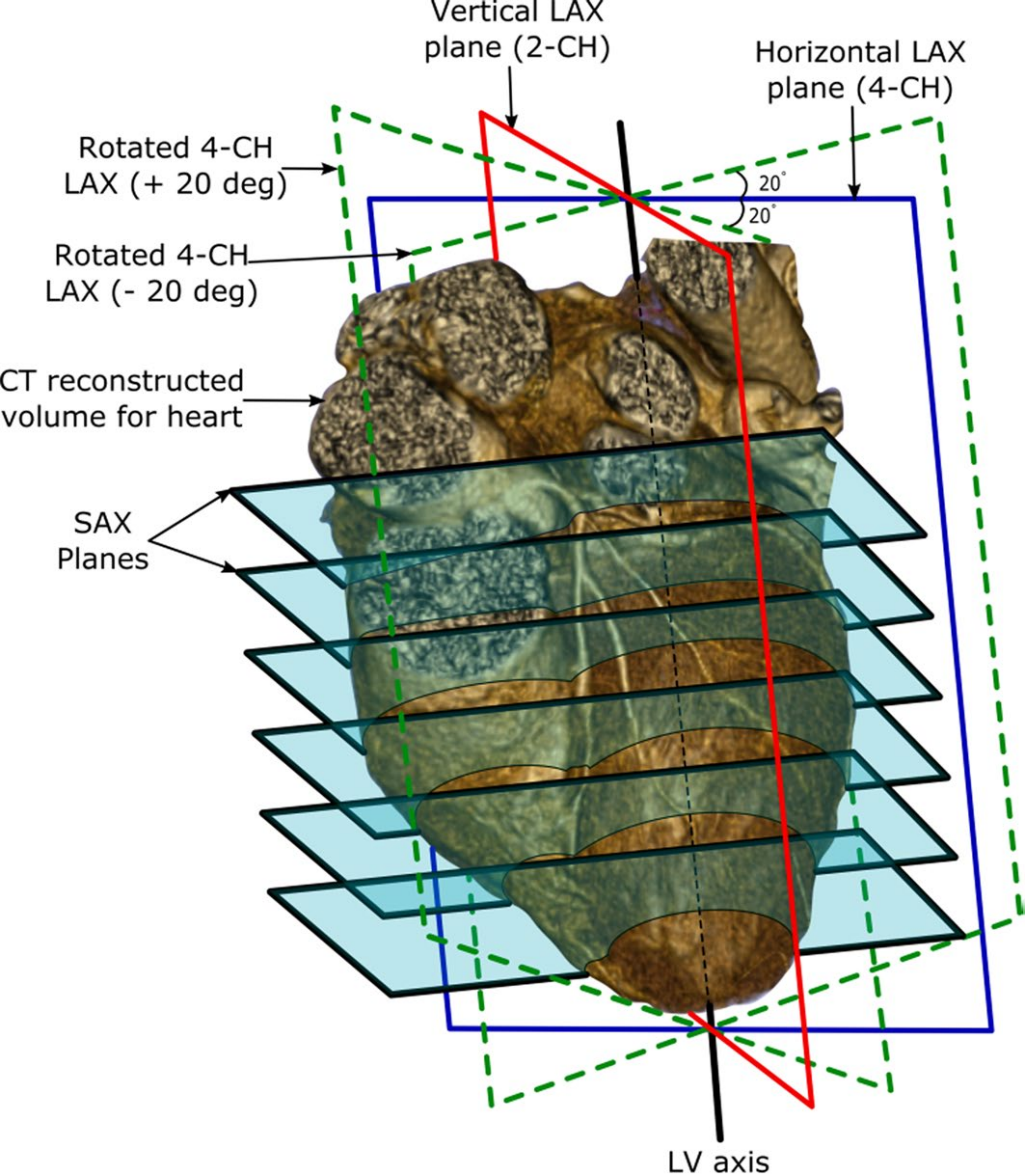
Cardiac CT reconstructed volume re-sliced to generate different cardiac cross-sections: SAX, horizontal LAX (i.e. 4-chamber), vertical LAX slice (i.e. 2-chamber), and two LAX slices (rotated ± 20° from the horizontal LAX plane)

In order to validate the developed model, the actual surface geometry of five human hearts have been constructed from data acquired using Computed Tomography (CT) as described in [18]. The dataset (publicly available on the internet [19]) contains single breath-hold cardiac-gated CT acquisitions with resolution 0.43 × 0.43 mm. Rendering of the 3D volume for each heart has been done and the volume is calculated and recorded as the ground truth. Then, each reconstructed volume was re-sliced to create cross-sectional images (matrix size: 512 × 512; voxel size: 0.43 × 0.43 × 3.5 mm) in the SAX and LAX directions as shown in Fig. 4. All processing was done using 3D-Slicer software tool [20]. First, a stack of twelve SAX slices covering the LV from base to apex was reconstructed. Secondly, a set of four LAX image slices with different orientations was reconstructed. The epicardium and endocardium contours of all acquired images have been manually delineated and used to calculate the difference LV volumes using the different methods.

Two sets of experiments have been done to test the performance and the robustness of the proposed method. The first experiment was done to quantify the error resulting from decreasing the number of SAX slices. In this experiment, the proposed model and mSimp method has been used to calculate the cardiac volume from one (4CH) LAX slice combined with different number of SAX slices (n = 4, 6, 8, 10, 12). The reduced set of SAX slices was selected such that we include the most basal slice in which the LV SAX contour appears as a complete ring. In addition, the set includes the most apical slice where the blood pool can barely be differentiated at end-systole phase. The remaining slices are selected to uniformly cover the distance between the already selected basal and apical slices. The volume estimated by each method was recorded and the mean and standard deviation of the error (relative to the ground truth) was calculated.

The second set of experiments was done to assess the robustness and reproducibility of the proposed method. First, the proposed method was tested to report its reliability in presence of misregistration among the LAX and SAX contours caused by respiratory motion. This was done by simulating different levels of breath-holds by randomly changing the location of the heart in the 3D space prior to the re-slicing operation described above. The breathing-induced motion was assumed to be in the superior-inferior direction with maximum displacement of 18 mm and in the anterior-posterior direction with maximum displacement of 2.5 mm [21]. The whole experiment is repeated 10 times with random displacement and the mean and standard deviation have been recorded for the different number of slices as above. Another experiment was done to test the reproducibility of the proposed model at different selections of LAX imaging planes. For this purpose, a set of LAX image planes was used to reconstruct: one horizontal LAX slice (i.e. 4-chamber view or 4CH); one vertical LAX slice (i.e. 2-chamber view or 2CH); and two rotated horizontal LAX slices (±20°) around the axis of the LV. Each of these four LAX images was combined with different numbers of SAX slices (*n* = 4, 6, 8, 10, 12) to calculate the volume.

### Model validation using real MRI data

A database of MRI images for 25 human subjects with symptoms of ischemic heart disease to test and evaluate the proposed model. Ten patients were scanned using 1.5T Siemens scanner, and 15 patients were scanned using 3T Philips scanner. The number of slices for each dataset was (9–12) SAX slices and one LAX slice. The pixel size was in the range of (1.116–1.406 mm) and the slice thickness ranges from 5 to 8 mm. Only the end-diastole and end-systole timeframes were considered for processing and analysis. In general, all slices are assumed to be acquired while the patient is holding his/her breath at the same level. To quantify the volume calculation error, the ground truth volume for a given heart was calculated by mSimp method applied to all available SAX slices. Then, the proposed model was applied to compute the volume using one LAX slice and different numbers of SAX slices: 1 (mid-cavity), 2 (most basal and most apical), 3, 5, 7, 9 and 11. For a number of slices >2, the slices are selected to include and uniformly cover the distance between the selected basal and apical slices. After calculating the volumes enclosed by the cardiac contours, two functional parameters, namely ejection fraction and stroke volume, have been estimated by the two methods and the error was calculated. Due to the anticipated inadequate performance of the mSimp method at very low number of SAX slices (<4), other model-based methods described in literature have been investigated and compared to the proposed method. These model-based methods approximate the shape of the heart using simple geometries such as single plane ellipsoid, Biplane ellipsoid, Teichholz model, Hemisphere cylinder (for more details about these models, please refer to [14]).

## Results and discussion

### Validation using CT-based phantoms

**Fig. 5:**
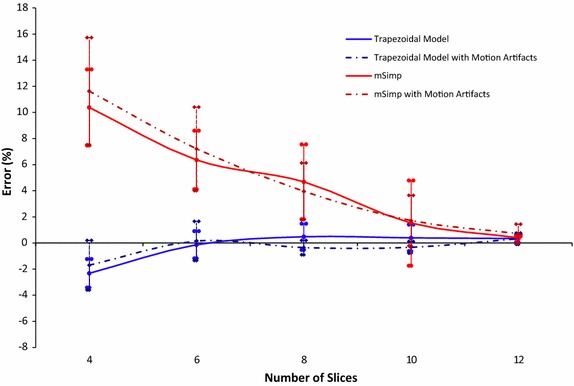
Error (mean ± SD) of the estimated volume at different number of SAX slices using the proposed method and the mSimp method (phantom experiment)

Figure 5 shows the results of the first phantom experiment, which measures the error in calculating the LV surface volume (LVV_s_) while increasing the number of slices from 4 to 12. As expected, the error of both the mSimp method (using *n* SAX slices) and the proposed trapezoidal model (using *n* − 1 SAX slices and one LAX slice) decreases with the number of slices. However, for the same number of slices, the error of the trapezoidal model is lower than that of the mSimp. At a small number of slices (<7), the figure shows that the error of the trapezoidal model (<−2.5 %) is much lower error than that of the mSimp (<10 %). At a higher number of slices, the error of the mSimp becomes less than 5 % and converges to 0.4 % error at maximum number of slices. On the other hand, the error of the proposed method remains almost constant for a number of slices more than seven with an overestimation of less than 0.5 %. Statistical analysis showed a statistically-significant difference (*p* value <0.01) between the errors of the two methods at all number of slices below eight.

Table 1 summarizes the results of the second set of experiments which measures the reproducibility of the proposed model when changing orientation of the LAX slice. It can be shown that no orientation leads to an error that is substantially and consistently lower than the errors of the other orientations. This might indicate that the proposed method is reliable to the specific selection of the LAX orientation. From another perspective, this shows that the proposed method has a lower bound on the error that cannot be further improved by changing the LAX slice orientation. Table 2 shows the error of both methods caused by simulated respiratory motion artifacts. Comparing these values to those reported in Fig. 5, it could be observed that the standard deviation of the error has increased due to the simulated movement. Nevertheless in both techniques, there was no significant difference between the reported errors before and after applying the respiratory motion.

**Table 1:** Percentage error (mean ± SD) of LV surface volume due to the reproducibility experiments

LAX orientation\#slices	4	6	8	10	12
4-CH	−2.3 ± 1.09	−0.13 ± 1.04	−0.48 ± 1.00	0.39 ± 1.01	0.34 ± 0.29
2-CH	−1.72 ± 1.14	0.11 ± 0.76	0.43 ± 1.15	0.57 ± 0.93	0.47 ± 1.14
+20 deg.	−1.2 ± 4.1	0.04 ± 1.01	0.32 ± 1.21	0.48 ± 0.98	0.63 ± 1.01
−20 deg.	−2.24 ± 1.4	−0.03 ± 0.95	0.34 ± 1.03	0.34 ± 0.89	0.03 ± 0.65

**Table 2:** Percentage error (mean ± SD) of LV surface volume due to the motion artifacts of the different simulated breath-holds experiments

Method\#slices	4	6	8	10	12
mSimp	11.63 ± 4.1	7.21 ± 3.2	3.96 ± 2.17	1.72 ± 1.93	0.72 ± 0.96
Trapezoidal model	−1.7 ± 1.9	0.16 ± 1.5	−0.35 ± 0.55	−0.33 ± 0.45	0.33 ± 0.43

### Validation using real MRI data

**Fig. 6:**
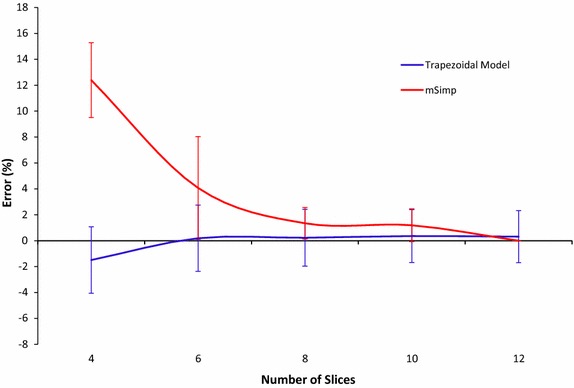
Error (mean ± SD) of the estimated volume at different number of slices using the proposed method and the mSimp method (real MRI data)

The results of the real data experiment show that the volume calculated by the trapezoidal model is generally lower than that of the mSimp method with statistically-significant lower error at number of slices less than 7. As can be shown in Fig. 6, the error of the trapezoidal model at 4 slices equals to −1.5 ± 2.56 % and keeps decreasing until it converges to 0.36 ± 2.04 % at higher number of slices. Similar to the phantom study, statistical analysis showed that the error of the proposed method in calculating the LV volumes is significantly lower than that of the mSimp with *p* value <0.01 for a number of slices less than eight.

**Fig. 7:**
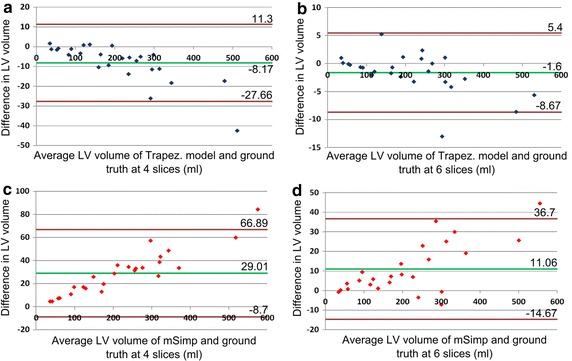
Bland-Altman plot for the LV volume calculation showing the agreement between the trapezoidal model and the ground truth at 4 and 6 slices (**a** and **b** respectively); and between the mSimp and the ground truth at 4 and 6 slices (**c** and **d** respectively)

To further illustrate the difference among the estimated volumes at low number of slices, Fig. 7 shows the Bland–Altman plot of the calculated LV volume using the different methods compared to the ground truth at 4 and 6 slices. As mentioned above, the ground truth is calculated by applying the mSimp method on the entire set of available SAX contours. As can be shown in Fig. 7a, b, the volume calculated by the proposed method comes in agreement with the ground truth with constant bias (independent of the LV volume) of −8.1 ± 9.9 ml at 4 slices and −1.6 ± 3.6 ml at 6 slices. On the other hand, as shown in Fig. 7c, d, the difference between the LV volume calculated by the mSimp and the ground truth depends on the LV volume. In particular, the mSimp has a mean bias of 29 ± 19.3 ml compared to the ground truth volume at 4 slices and 11 ± 13.1 ml at 6 slices. This indicates the accuracy of the proposed method, relative to the mSimp method, to calculate the LV volume when only a small number of slices are acquired.

The calculations of the ejection fraction (EF), stroke volume (SV), and myocardial LV mass (LVM) of each dataset are listed in Table 3. As can be seen in the table, the average error of calculating the EF error in both methods is less than 1.55 % for all number of slices with a SD value which decreases with increasing the number of slices. Analysis showed no statistically-significant difference between the two methods. On the other hand, the error of calculating the SV and myocardial LVM was found significantly lower (*p* value ≈ 0) in the proposed method at number of slices less than seven.

**Table 3:** Percentage error (mean ± SD) of LV surface volume (LVV_s_), EF, SV and myocardial LV mass (LVM) computed by the proposed Trapezoidal and the mSimp methods calculated at different numbers of slices 4, 6, 8, 10 and 12

Method	# slices	4	6	8	10	12
Trapezoidal model	LVV_s_	−2.3 ± 1.09	−0.13 ± 1.04	−0.48 ± 1.00	0.39 ± 1.01	0.34 ± 0.29
	EF	0.44 ± 4.1	0.77 ± 1.7	−0.25 ± 1.44	0.2 ± 0.9	0.21 ± 0.87
	SV	−2.7 ± 9.1	−0.16 ± 2.7	−0.7 ± 2.5	0.18 ± 0.83	0.21 ± 0.92
	LVM	−3.46 ± 7.8	0.86 ± 3.57	0.79 ± 3.2	0.98 ± 2.59	0.92 ± 2.38
mSimp	LVV	12.4 ± 2.8	4.1 ± 3.9	1.4 ± 1.3	1.19 ± 1.26	0.0 ± 0.0
	EF	−0.98 ± 7.31	1.53 ± 2.17	−0.67 ± 1.45	−0.018 ± 0.07	0.0 ± 0.0
	SV	7. 8 ± 12.53	4.24 ± 5.64	0.69 ± 2.35	−0.06 ± 0.22	0.0 ± 0.0
	LVM	16.8 ± 5.07	5.57 ± 4.99	2.47 ± 2.43	2.42 ± 2.56	0.0 ± 0.0

At extremely small number of slices (three slices or less), the performance of the proposed method was compared to different models that were proposed in literature to handle the problem of severely reducing the number of slices. Table 4 shows the percentage error of calculating the LV surface volume using these models compared to the proposed model at the same number of slices. As can be seen in the table, using two SAX slices, the Biplane ellipsoid and Hemisphere cylinder models resulted in an error of −9.9 ± 5.88 % and 3.6 ± 7.4 % respectively. This error is significantly higher than that of the proposed trapezoid model (=1.92 ± 5.96 %) using one LAX and one SAX slice. At three slices (2 SAX and 1 LAX), the modified Simpson method resulted in an error of −5.73 ± 8.95 % compared to −2.28 ± 4.38 % resulting from the proposed method. Nevertheless, it was found that at such very small number of slices, the error of the other functional parameters increases significantly relative to the error at 4 slices. For example, the LVM and SV were found to be −18.1 ± 11.9 and −16.68 ± 10.1 respectively at 2 slices, which may not be appropriate for accurate estimation of the cardiac function.

**Table 4:** Percentage error (mean ± SD) of LV surface volume using different models that using either two, or three slices, and the corresponding Trapezoidal model at the same number of slices

Number of slices	Type of slices	Model name	Error (mean ± SD)	Trapezoidal model
2 slices	2 LAX	Biplane ellipsoid	−9.9 ± 5.88	1.92 ± 5.96
	1 SAX + 1 LAX	Hemisphere cylinder	3.6 ± 7.4	
3 slices	2 SAX + 1 LAX	Modified Simpson rule	−5.73 ± 8.95	−2.28 ± 4.38

One advantage of the proposed method is the simplicity of the computations given by Eq. (7). The equation involves only a computation of the area of three contours (or polygons) in addition to the length of two line segments. That is, combining the information from the LAX and SAX views does not involve actual handling of the 3D positions of the SAX or the LAX contour points. However, it is worth mentioning that an implicit step is required to compute the intersection line between the LAX plane and each SAX plane. The overall average computation time on a PC (Dual-core 3 GHz processor, 4 GB RAM) using Matlab implementation (Mathworks, Inc.) is 32 ms per imaging cross-section.

## Conclusion

In this work, a method for estimating the left ventricular volume from segmented MRI images has been presented. The method incorporates cardiac long axis and short axis cross-sectional views to accurately estimate the myocardium volume. A simple trapezoidal model was used to approximate the myocardium LAX cross-section between pairs of SAX slices. This allowed accurate estimation of the volume compared to the traditional techniques. Results on simulated and real MRI datasets showed the superiority of the proposed method compared to other available methods at small number of slices.

## Abbreviations

LV: left ventricle
MRI: magnetic resonance imaging
CT: computed tomography
SAX: short axial
LAX: long axial
mSimp: modified Simpson’s method
3D: three dimensional
4CH: 4-chamber
2CH: 2-chamber
LVV_s_: left ventricular surface volume
EF: ejection fraction
SV: stroke volume
LVM: left ventricular mass
SD: standard deviation
PC: personal computer
